# Marginalization and Precariat: The Challenge of Intensifying Life Construction Intervention

**DOI:** 10.3389/fpsyg.2016.00444

**Published:** 2016-03-30

**Authors:** Annamaria Di Fabio, Letizia Palazzeschi

**Affiliations:** Department of Education and Psychology (Psychology Section), University of FlorenceFlorence, Italy

**Keywords:** marginalization, precariat, life construction intervention, decent work, case study, Life Adaptability Qualitative Assessment (LAQuA), Career Counseling Innovative Outcomes (CCIO)

## Abstract

This article discusses the case study of a graduate student who, at the time of the study, was doing an internship, considered in the literature as a new form of precariat (temporary or insecure employment). The student participated in a life construction intervention during which he completed two new qualitative instruments: the Life Adaptability Qualitative Assessment (LAQuA) and the Career Counseling Innovative Outcomes (CCIO) before and after the life construction intervention. The results are discussed in the article. The life construction intervention helped the participant understand himself better, develop his life and career paths, and construct his identity. The study confirmed the value of enhancing life construction interventions using a preventive approach, particularly for precarious people (people in temporary or unstable jobs), with early interventions starting with young internees in organizations.

## Introduction

The concept of marginality first appeared in the field of sociology in the early 20th century, more particularly in Park’s (1928) essay, “Human migration and the marginal man.” The “marginal man” is someone “on the margin of two cultures and two societies which never completely [interpenetrate and fuse]” (Park, 1928, p. 892). He is someone with “spiritual instability, intensified self-consciousness, restlessness, and malaise” (Park, 1928, p. 893). The concept of marginality is important in sociological thinking and has a multiplicity of meanings (Billson, 1996). Since 1928, there have been three forms of marginality: cultural marginality, which is determined by differences in terms of race, ethnicity, religion, and other cultural indicators; social marginality, which occurs when an individual is not considered part of a positive reference group owing to age, timing, situational constraints, or occupational role; and structural marginality, which results from the political, social, and economic powerlessness of specific disadvantaged groups in societies (Billson, 1996).

In the psychology field, a shift has occurred from sociological marginalization to social psychology marginalization (Young, 2000; Mullaly, 2007). Young (2000) maintains the most dangerous form of oppression occurs when the labor market does not enable everyone to have a job. On an individual level, this kind of exclusion prevents people from participating meaningfully in society (Young, 2000). Mullaly (2007) contends that minority groups (e.g., those with disabilities, women, racial minorities, elderly individuals) can experience marginalization due to dominant discourses in society. In community psychology, marginalized people have little control over their lives and available resources, limited opportunities to make social contributions, low self-confidence, and low self-esteem (Burton and Kagan, 2003). Marginalized people are also often stigmatized, leading to a vicious circle marked by a lack of supportive relationships and the ability to participate in community life, resulting in further isolation (Burton and Kagan, 2003).

A marginalized workforce was recently analyzed by the Society for Industrial and Organizational Psychology (SIOP; Maynard and Ferdman, 2015). In the analysis, the workforce defined what it meant to be marginalized, stressing the exclusion from access to power and resources and being on the periphery of society (Maynard and Ferdman, 2015). Marginalized workers are, generally speaking, categorized as including the working poor, immigrant/migrant workers, young workers (school leavers, victims of child labor), chronically unemployed individuals, groups that have minority or lower social status (e.g., ethnic minorities, older workers, workers with disabilities), and victims of human trafficking (Maynard and Ferdman, 2015). Marginalized workers are also defined by the work they do as revealed by recent research on “dirty work” (Bergman and Chalkley, 2007) including temporary/contract, seasonal, and intermittent work (Connelly and Gallagher, 2004; Ashford et al., 2007). Marginalized workers face many difficulties: cultural differences, low motivation and self-efficacy, difficulty in accessing organizational resources, difficulty in identifying and taking advantage of developmental opportunities, and work-family conflicts (Maynard and Ferdman, 2015). Industrial and organizational psychological research has not adequately covered the diversity of the working population, particularly marginalized workers, therefore making it necessary to carry out more research on this category of workers (Maynard and Ferdman, 2015). Industrial and organizational psychologists can help marginalized workers meet the challenges they face by “(a) assisting with social and organizational assimilation and conflict prevention/resolution; (b) promoting coaching, mentorship, career development, and job initiatives; (c) finding ways to increase acceptance of these groups within the organization; (d) identifying factors that reduce the real or perceived risk in hiring workers from traditionally marginalized groups” (Maynard and Ferdman, 2015). These steps can help merge business needs and interests with the needs and talents of marginalized workers (Maynard and Ferdman, 2015).

The precariat can be defined as a form of worker marginalization in the 21st century. The ILO (2015) comments on the changing nature of the world of work and reports that global unemployment figures reached 201 million in 2014, over 30 million higher than before the start of the global crisis in 2008. The challenge is to provide jobs to the more than 40 million additional people who enter the global labor market every year. Employment relationships are also becoming less stable in terms of the ILO’s standard employment model (ILO, 2015). Policies need to be adapted to the changing nature of work just as labor norms need to be adapted to the new and different kinds of employment (ILO, 2015).

In the post-modern era, characterized by globalization and more flexible labor relations, the number of people with insecure jobs is increasing (Guichard, 2013). Standing (2014) states that many working people today are experiencing “a precarious existence [precariat]. Friends, relatives, and colleagues would also be in a temporary status of some kind, without assurance that this was what they would be doing in a few years’ time, or even months or weeks hence. Often they were not even wishing or trying to make it so” (Standing, 2014, pp. 6–7). The term precariat was first introduced by French sociologists in the 1980s in reference to temporary or seasonal workers. In Italy, the term “precariato” means not only people with temporary work but also people with a “precarious” existence, whereas in Germany the term denotes people without job who have difficulty in integrating socially and not only temporary workers (Grimm and Ronneberger, 2007). In Japan, precariat is synonymous with “the working poor,” but it refers also to young activists who fight to obtain better working and living conditions (Obinger, 2009). Standing (2014) identifies five kinds of precariat: (1) precariat as those with limited citizenship rights; (2) precariat as those having temporary jobs; (3) precariat as those in part-time employment; (4) precariat as those who work in call centers; (5) precariat as those working as interns. This last form of precariat can serve as a channel for conducting young people into other precariats. Alongside the precariat is the concept of peripheral workers (Guichard, 2009), that is, marginalized workers. These forms of precarious and insecure work do not allow people to build proper identities and careers (Guichard, 2009).

The foregoing indicates the need for life construction intervention among the different types of precariat workers in order to promote sustainable decent work. A preventive approach (Hage et al., 2007; Kenny and Hage, 2009; Di Fabio and Kenny, 2015; Di Fabio et al., in press) stresses the importance of building the strengths of individuals (Di Fabio and Palazzeschi, 2008a,b, 2009, 2012; Di Fabio and Blustein, 2010; Di Fabio and Kenny, 2012a,b; Di Fabio et al., 2012, 2013, 2014; Di Fabio and Saklofske, 2014a,b; Di Fabio, 2014b, 2015a). Life construction intervention should be administered to precarious people in particular, with early interventions starting with the earliest form of precariat (i.e., precariat as the internship of young people in organizations) and moving to all other forms of precariat.

This article discusses the case study of a graduate student who, at the time of the study, was doing an internship (considered in the literature as a new form of precariat) and who participated in a life construction intervention.

### Aim of the Case Study

The case study describes the process and usefulness of a life construction intervention in helping the research participant (client) to better understand himself, to develop his life and career paths, and to construct his own identity. The case study is characterized by working with a unique participant in a one-to-one research setting and it is based on a qualitative, interpretive paradigm (Patton and McMahon, 1999). The case study enhances participant’s involvement in his process of life construction facilitating the process of construction, deconstruction, reconstruction, and co-construction of his life story (Savickas, 2011; Maree, 2014). The case study also helps in describing the changes related to the intervention. The client of this case study is a graduate student who was doing an internship, considered in the literature as a new form of precariat (Guichard, 2009; Standing, 2014) because it is temporary or insecure employment. This case study is a way to deepen process involved in life construction intervention in a precariat situation.

The study attempted to answer the following two research questions.

• How was the life construction intervention administered to a young Italian man with a degree in forest and environmental sciences and who was doing an internship (considered a new form of precariat)?
• How did the life construction intervention help the young man to enhance his self-awareness, to develop his life and career paths, and to construct his own identity?

## Materials and Methods

### Participant and Context

The participant in the study, Christian (a pseudonym), was a young man who had graduated in forest and environmental sciences at the University of Florence in Italy and was doing an internship at the Municipality of Florence for 6 months to monitor the state of the trees in the city. He asked to participate in a life construction intervention at the career counseling center of the university. Christian was 23 years old at the time of the study and requested the intervention because he was not sure about what career he should follow or whether he should do a second-level Master’s degree or other postgraduate course.

### Qualitative Measures

#### Life Adaptability Qualitative Assessment (LAQuA)

The Life Adaptability Qualitative Assessment (LAQuA; Di Fabio, 2015b) is a new qualitative instrument developed to qualitatively assess the effectiveness of life construction interventions. In particular, this instrument evaluates adaptability, assessing change or lack of change in individuals’ lives in narratives before and after the intervention. The LAQuA consists of 12 written questions with three questions for each dimension (Concern, Control, Curiosity, Confidence) of the Career Adapt-Abilities Inventory – International Version 2.0 (Savickas and Porfeli, 2012). The 12 written questions are the following:

Concern: (1a) What does it mean to you to be oriented toward your future? (1b) Do you think you are oriented toward your future? (1c) Why?

Control: (2a) What does it mean to you to take responsibility for your future? (2b) Do you think you do take responsibility for your future? (2c) Why?

Curiosity: (3a) What does it mean to you to be curious about your own future? (3b) Do you think you are curious about your future? (3c) Why?

Confidence: (4a) What does it mean to you to have confidence in your own ability to build your future? (4b) Do you think you have confidence in your ability to build your future? (4c) Why?

The comparison of the answers to the 12 questions before and after the life construction intervention is done using 24 qualitative indicators in respect of each of the four dimensions (Concern, Control, Curiosity, and Confidence) of the Career Adapt-Abilities Inventory – International Version 2.0 (Savickas and Porfeli, 2012), which is structured in the LAQuA coding system. The LAQuA coding system detects change or lack of change for each dimension of Adaptability at different levels of reflexivity (Increased Reflexivity, Revised Reflexivity, Open Reflexivity, Enhanced Reflexivity, and No change).

#### Career Counseling Innovative Outcomes (CCIO)

The Career Counseling Innovative Outcomes (CCIO; Di Fabio, 2016) is a new qualitative instrument that assesses life construction intervention outcomes and evaluates the effectiveness of interventions. The CCIO was inspired by the innovative moments coding system used in psychotherapy (Gonçalves et al., 2011) and its application in career construction counseling (Cardoso et al., 2014). Whereas the innovative moments coding system is used to monitor the process of change during psychotherapeutic intervention (Gonçalves et al., 2011) and career construction counseling intervention (Cardoso et al., 2014), the CCIO was developed specifically to analyze narratives before and after life construction interventions. The CCIO consists of seven questions developed on the basis of the narrative paradigm (Savickas, 2011) that are asked before and after the intervention: (1) In which ways can this intervention be useful/was this intervention useful to you? (2) What are your most useful resources? (3) What are the main obstacles you encounter? (4) Who do you think can be useful to you? (5) What do you think can be useful to you? (6) What are the main challenges you face? (7) What are the main objectives you are hoping to achieve?

The narratives elicited by these seven questions are coded using the five categories system developed by Gonçalves et al. (2011). The five categories are: Action, Reflection (type I and type II), Protest (type I and type II), Reconceptualization, Performing change.

### Procedure

The LAQuA and the CCIO were administered before and after the life construction intervention, actually, a life meaning intervention (Bernaud, 2015). The LAQuA and CCIO were administered by a psychologist trained in the administration of these two qualitative instruments. The participant’s initial and subsequent responses to the written questions of these two narrative instruments were compared by three independent, trained expert reviewers (raters). An interrater reliability analysis using the Kappa statistic was carried out to establish the level of consistency among the raters.

The study was conducted according to Italian laws on privacy and informed consent (Law Decree DL-196/2003), which are in line with the latest version of the Declaration of Helsinki revised in Fortaleza (World Medical Association [WMA], 2013).

The participant (Christian) took part in a life meaning intervention (Bernaud, 2015) divided into three 1-day sessions (8 h a session) in a group context using the modality of the power of an audience. The aim of life meaning interventions is to permit participants to ask themselves questions about the meaning of their lives both at work and outside work thereby offering them new awareness on how to construct their lives.

### Criteria for Quality Assurance

The application of the following quality assurance criteria is fundamental to guarantee the trustworthiness of the results using of different modalities for the data collection and analysis: credibility, confirmability, transferability, and dependability in the data collection and analysis process can help guarantee the trustworthiness of research results (Maree, 2012). Credibility of data refers to “factors such as the significance of results and their credibility for participants and readers” (Maree, 2012, p. 141). Credibility in the present study was guaranteed by external verification of the results by other researchers. Confirmability refers to “the objectivity of the data and the absence of research errors. Results can be regarded as confirmable when they are derived from the participants and the research conditions rather than from the (subjective) opinion of the researcher” (Maree, 2012, p. 142). Confirmability was assured by external researchers who assessed whether the methods and procedures of the study had been described sufficiently clearly to enable verification. Transferability refers to “the extent to which the results can be ‘exported’ and generalized to other contexts” (Maree, 2012, p. 142). Transferability in the present study was obtained through the accurate description of the participant’s situation and the methodologies used to obtain the narratives. Detailed information was given also on the context of the study to enable other researchers to evaluate the applicability of the results to other contexts. Dependability refers to “the stability and consistency of the research process and methods over time and influences the degree of control in a study” (Maree, 2012, p. 141). Dependability was assured by the independent analysis of the participant’s narratives by three expert raters.

## Results

Below are given the participant’s responses to the written questions of the LAQuA before and after the life construction intervention as well as the results of the analysis obtained through the LAQuA qualitative indicators and the different levels of reflexivity (Increased Reflexivity, Revised Reflexivity, Open Reflexivity, Enhanced Reflexivity, and No change).

The participant’s response to the first LAQuA question before the life construction intervention was: “*To me to be oriented toward my future means considering that the choices that I make today can shape my future*”^1^ (qualitative descriptor: Predicting); and after the life construction intervention it was: “*To me to be oriented toward my future means to pay attention to the choice I’m taking in this period of my life. After the intervention, I confirmed that my field of interest is forest sciences. Also my internship experience is allowing me to be more aware of this interest. I understand also that I need to extend my knowledge and competence in forest sciences, so I want to find a specialized university course in this field. I want to begin to gather information on a second-level Master’s degree and post-university specialized courses and then evaluate the different possibilities*” (identical qualitative descriptor but more in-depth reflexivity: Predicting; Increased reflexivity: in the narratives produced after the life construction intervention, there were identical descriptors, but they were presented with more in-depth reflexivity).

The participant’s response to the second LAQuA question before the life construction intervention was: “*To me, to take responsibility for my future means taking decisions by myself, without being influenced by others, particularly by my parents*” (qualitative descriptor: Autonomous); after the life construction intervention it was: “*To take responsibility for my future means deciding by myself on what I want to do in my future. My parents don’t understand that it is important for me to continue studying, but I think that my preparation is not sufficient to enter the world of work. I understand this also thanks to my internship experience*” (identical qualitative descriptor but more in-depth reflexivity: Autonomous). “*If my parents don’t financially support my choice to continue studying, I will find some work to pay for my training*” (new, different qualitative descriptor: Responsible). “*I will do what I think it is right for me*” [new, different qualitative descriptor: Honest; Enhanced reflexivity (E): in the narratives produced after the life construction intervention, there is an identical descriptor/s but presented with more in-depth reflexivity plus a new, different descriptor/s].

The participant’s responses to the third LAQuA question before the life construction intervention was: “*To me, to be curious about my future means gathering information about postgraduate training possibilities in relation to forest sciences and evaluating positive and negative aspects of each different option before choosing*” (qualitative descriptor: Inquisitive); after the life construction intervention it was: “*To be curious about my future means informing myself about postgraduate courses in forest sciences by analyzing different options before making a choice* (identical qualitative descriptor: Inquisitive) *and observing different ways of doing things during my internship as a way to learn from direct experience* [new, different qualitative descriptor: Recognizing/Discovering; Open reflexivity (O): in the narratives produced after the life construction intervention, there is an identical descriptor/s – with the same level of reflexivity in presenting the descriptor – plus a new, different descriptor/s].

The participant’s response to the fourth LAQuA question before the life construction intervention was: “*To me, to have confidence in my own abilities to build my future means believing I can overcome the difficulties and the obstacles that I could encounter during my path*” (qualitative descriptor: Resilient); after the life construction intervention it was: “*To have confidence in my own abilities to build my future means to be engaged in learning new knowledge and skills. I think it is fundamental to become increasingly more specialized in the field of forest science*” [new, different qualitative descriptor: Innovative; Revised reflexivity R: in the narratives produced after the life construction intervention, the previous descriptor/s has disappeared, and a new, different descriptor/s has appeared].

Below are shown the participant’s responses to the seven questions of the CCIO before and after the life construction intervention as well as the results of the analysis in terms of the five categories (Action, Reflection, Protest, Reconceptualization, and Performing change) of the CCIO coding system (Di Fabio, 2016).

The participant’s response to the first CCIO question before the life construction intervention was: “*I hope that this intervention will help me better understand what I want to do in this period of my life. I have graduated in forest and environmental sciences, and I’m doing an internship at the Municipality of Florence for six months to monitor the state of the trees in the city. I don’t know what I really would like to do now, particularly whether I should do a second-level Master’s degree or a postgraduate course, or whether it would be better to begin working immediately*”; after the life construction intervention it was: “*This intervention was useful to me because it allowed me to clarify what I really want to do. I understood not only that the activities that I’m doing during my internship are interesting to me, but above all I understood that preserving the trees and the nature in general has a profound sense for my life because it also constitutes an important value for me*” (Reflection Type II IM). “*Before the intervention, I was not sure that working to protect trees and forests was the right choice for me, because I always thought that this choice was the choice that my parents made for me and so not completely my choice, now I realize that the care of trees and nature is what I really want to do because it is fully in line with my ideals*” (Reconceptualization IM).

The participant’s response to the second CCIO question before the life construction intervention was: “*Actually, I don’t know precisely what my resources are*”; after the life construction intervention it was: “*When I began this intervention one of my principal concerns was that I did not have sufficient knowledge and skills to start a profession as an agricultural consultant. Furthermore, I wasn’t sure if working in the field of agricultural or forest sciences would really give me a satisfactory life. During the intervention, I started to think about using my internship in a different way, and I asked my supervisor about the knowledge and skills that I had, and if he could indicate the best way to improve them. Now I was reassured and more confident in myself also recognizing the need to find a specialized postgraduate path and to gather information about different possibilities”* (Action IM). “*Furthermore, in particular, the intervention made me aware of my principal resource in terms of my personal awareness that I have a mission to protect plants and nature. This mission gives a sense to my life and it is also important for my work*” (Reflection Type II IM). “*So I’m starting with the new project of continuing my studies in the field of forest sciences. It is a project that I had thought of abandoning because my parents wanted me to find a job immediately*” (Performing change IM).

The participant’s response to the third CCIO question before the life construction intervention was: “*I have two principal obstacles. The first obstacle relates to the fact that I’m unable to decide if I would like to start working immediately, or if I would like to continue studying. The second obstacle relates to the fact that my parents are pressing me to start to work immediately, and they probably won’t support me financially if I continue to study*”; after the life construction intervention it was: “*I understood that I have to worry less about the opinions of other people. I always listened a lot to the opinions of my parents, but now it is time to build my life on my own*” (Protest Type I IM). “*Now that I really understand that the choice to study forest sciences was not casual and that it is linked to important values that I sincerely believe in, I don’t intend to give up because life satisfaction is crucial to me*” (Protest Type II IM). “*Now I’m starting to gather information about various second-level Master’s degrees or postgraduate courses so that I can evaluate different possibilities and make a choice*” (Action IM).

The participant’s response to the fourth CCIO question before the life construction intervention was: “*I think you* [the life construction counselor] *could be useful to me by helping me understand what I really would like to do; if I would like to continue studying by doing a Master’s degree or a postgraduate course, or if it would be better to begin to work immediately*”; after the life construction intervention it was: “*If before the intervention I thought that you as a professional could be useful to me to give me advice on my future, now I understand that you were useful to me because you helped me become aware of what I really would like to achieve in my life*” (Reconceptualization IM). “*My internship supervisor was also useful to me because he helped me realize that in the future I would prefer to work as an agricultural technician in a public institution rather than as an agricultural freelance consultant. In my life, I would like to make a contribution to my community and to environmental protection*” (Performing change IM).

The participant’s response to the fifth CCIO question before the life construction intervention was: “*I think that it can be really useful to me to understand if I would like to continue to study by doing a Master’s degree or a postgraduate course, or if it would be better to begin working immediately*”; after the life construction intervention it was: “*I think that it can be really useful to me to gather information on Master’s degrees or postgraduate courses related to forest sciences, and also to speak to professionals working in this field to better understand the lives they lead, and then to determine how to construct my life*” (Action IM). “*After collecting this information, I can better evaluate which path will help me to realize myself most fully. I’m also thinking of the possibility of paid internship, similar to what I’m doing at the Municipality of Florence. I could then study and work at the same time without having to ask my parents for help. Even if the internship can be considered a precarious working condition, this kind of experience will help me understand if I would really like to continue studying to enter the profession I desire*” (Performing change IM).

The participant’s response to the sixth CCIO question before the life construction intervention was: “*The main challenge that I feel I’m facing at the moment is to understand what I really what to do, what kind of job really satisfies me, and if it is possible to obtain this kind of job in the current economic crisis. Understanding all these things is necessary for me to construct my future life*”; after the life construction intervention it was: “*Before the intervention I was confused about what I really wanted to do in my life; after the intervention I’m sure that I would like to work in the field of the forest sciences and that I would like to continue studying in this field*” (Reconceptualization IM). “*Making this choice of continuing to study won’t necessarily disappoint my parents who may perhaps understand the importance of this choice for my life*” (Reflection Type II IM).

The participant’s response to the seventh CCIO question before the life construction intervention was: “*The main objective that I hope to achieve is to clarify what I want to do in my life*”; after the life construction intervention it was: “*Now my main objective is to finish this period of internship at the Municipality of Florence, to gather information on Master’s degrees or postgraduate courses in the forest sciences field, and to look for other possibilities of internship to earn money to pay for my studies*” (Action IM). “*I think that even if paid internship is a form of precarious work, it gives me an opportunity to earn money for my studies to help me construct a more significant life and also to gain experience in different contexts*” (Reflection Type II IM). “*My main objective in my life is to realize my mission of protecting plants and nature through my work*” (Reflection Type II IM).

## Discussion

This case study showed the value of enhancing life construction intervention using a preventive approach (Hage et al., 2007; Kenny and Hage, 2009; Di Fabio and Kenny, 2015; Di Fabio et al., in press) toward precarious (vulnerable) people, beginning with early interventions for young internees in organizations. Internship can be considered a first form of precariat work that can have consequences for the construction of a desirable identity and a desirable career (Guichard, 2009; Standing, 2014). Helping young people during the transitions in their early careers in the 21st century means promoting positive career outcomes and decency in their lives. Life construction counseling (Guichard, 2013) is an intervention specifically developed to help people cope with transitions and design new chapters in their lives (Savickas, 2011). In this case study, the evolution of the participant could be seen through the analysis of the narratives before and after the career construction intervention by means of the LAQuA (Di Fabio, 2015b) and the CCIO (Di Fabio, 2016).

The LAQuA analysis revealed changes in the participant’s reflexivity regarding adaptability in the narratives before and after the life construction intervention. The increase in the level of reflexivity applied to all four dimensions of adaptability (Concern, Control, Confidence, and Curiosity). Regarding Concern, the participant realized that today’s choices could determine his future; regarding Control, the participant understood that it was important to make decisions by himself and to trust himself; regarding Curiosity, the participant realized the relevance of investigating different options before choosing a particular post-degree study course and of observing different ways of doing things, particularly during his internship; regarding Confidence, the participant realized the importance of learning new skills and of being able to overcome obstacles in constructing his life and career (Di Fabio, 2015b; Sartori et al., 2015).

The analysis using the CCIO of the five dimensions: Action, Reflection, Protest, Reconceptualization, and Performing revealed changes in the participant’s narratives before and after the life construction intervention. This was seen in terms of Action where he actively explored solutions (Gonçalves et al., 2011; Di Fabio, 2016) by gathering information on Master’s degrees and other postgraduate courses on forest sciences, by speaking with professionals working in this field to better understand their lives, and by looking for other internship possibilities to earn money to pay for his studies. There were also changes in his narratives in terms of reflection regarding the choice to continue studying by doing a second-level Master’s degree or other postgraduate course or to begin working immediately and to come to terms with his new emerging identities (Gonçalves et al., 2011). The participant engaged in adaptive self-instruction after his increased awareness that preserving trees and nature had a profound value for him. The main objective of his life was therefore to realize his mission of protecting plants and the environment through his work. The analysis of the narrative also raised the issue of the difference between what a person really wants to do compared to what others would like him to do in his life, the so-called protest innovative moment (Gonçalves et al., 2011; Di Fabio, 2016). Two types of “protest innovative moments” emerged from the analysis. The first was the participant’s awareness that it was now the time to build his life himself and to stop following the wishes of his parents. The second was his stated intention to continue studying forest sciences because it was in line with his authentic self and not just a casual choice for him (Di Fabio, 2014c).

The analysis of the narratives also revealed a “reconceptualization innovative moment” concerning the transition between two positions (past and present; Gonçalves et al., 2011; Di Fabio, 2016). The participant went from a position where he did not know if really wanted to continue studying forest sciences to a position of greater awareness of the importance of his mission to protect plants and nature because in this way he could give sense to his life and construct his career path accordingly. Finally, the analysis of the narratives revealed a “performing change innovative moment” in terms of the participant’s investment in new projects as a result of the change process (Gonçalves et al., 2011; Di Fabio, 2016). More particularly, he was thinking of looking for other possibilities of paid internship so that he could study and work at the same time without having to ask his parents for help. Even if the internship could be considered a “precarious employment condition,” after the life construction intervention, the participant described this kind of precariat as a way to acquire new competences and to understand if he really liked the job he was doing and also if he really wanted to continue studying to enter the profession and live the life he desired. The internship experience also made him realize that, because of his desire to serve his community, he would prefer to work as an agricultural technician in a public institution rather than as an agricultural freelance consultant. Overall, the analysis of the narratives before and after the life construction intervention indicated changes in the narratives, showing that this kind of intervention enabled the participant to understand himself better, to develop his life and career paths, and to develop his purposeful identitarian awareness (Di Fabio, 2014c) and authentic self (Di Fabio, 2014c).

The case study also showed that through life construction intervention, internship (considered a form of precariat, Standing, 2014) could be transformed into a framework where young people could gain new knowledge and competences, could experience new contexts and different roles, could prove their interests, could individuate role models to construct a desirable identity and, consequently, a desirable life and career. It seems that early life construction interventions for young people doing internships could obviate the negative consequences of precariat such as difficulties in constructing a desirable identity and a desirable career (Guichard, 2009; Standing, 2014).

Although this study showed changes in the narratives produced by the participant before and after the life construction intervention, the effectiveness of the intervention needs to be confirmed by further studies and research with larger samples. The trustworthiness and credibility of the study were guaranteed, but a limitation could be the subjective interpretation of the researchers. A follow-up session 6 weeks after the intervention showed that the participant’s new intentions were being implemented as he had started to gather information on Master’s degrees and other postgraduate courses in the forest sciences field and to look for other internship possibilities to earn money to pay for his studies himself. He had also spoken to his parents about the possibility of studying again, and they agreed, especially if he would be able to pay the studies himself. Nevertheless, a follow-up assessment 6–12 months after the life construction intervention would be useful to confirm the results obtained in the study.

Despite the mentioned limitations, the study did indicate the value of enhancing life construction intervention using a preventive approach, particularly for young people doing internships to help them construct desirable identities and lives. Life construction interventions can be expanded to all forms of precariat from internships done by young people in organizations to workers in call centers. The extension of life construction interventions to all “precarious workers” could help them construct more desirable identities promoting self-realization (Blustein, 2006) and the development of a purposeful identitarian awareness (Di Fabio, 2014c) based on the authentic self (Di Fabio, 2014c). “Decent” work could be enhanced by activities experienced as authentic and motivating (Blustein, 2006) and also by nutritive relations at work (Blustein, 2011; Di Fabio, 2014a), preventing marginalization and precariat. This can be achieved with the assistance of counselors who can identify and develop the talents in young people on the threshold of early careers, thereby helping them enhance their self-awareness, self-efficacy, and employability.

## Author Contributions

AD conceptualized the study and choose the theoretical framework. AD and LP qualitatively analyze the case study. AD and LP wrote methods and results. Then the two authors wrote the paper together and read and revised the manuscript several times.

## Conflict of Interest Statement

The authors declare that the research was conducted in the absence of any commercial or financial relationships that could be construed as a potential conflict of interest.

## Footnotes

1 The verbatim responses of the participant have been lightly edited to preserve their authenticity.
